# Massive gastrointestinal hemorrhage caused by Henoch-Schoenlein purpura: A case report

**DOI:** 10.1097/MD.0000000000028240

**Published:** 2021-12-17

**Authors:** Shuo Wang, Hongyan Tang, Wei Du, Yiyi Ding

**Affiliations:** The First People's Hospital of Changde City, Changde, Hunan, China.

**Keywords:** Children, gastrointestinal bleeding, Henoch-Schoenlein purpura

## Abstract

**Rationale:**

Henoch-Schoenlein purpura (HSP) is a systemic small-vessel vasculitis that commonly occurs in children. Gastrointestinal HSP can rarely progress to gastrointestinal perforation, followed by massive gastrointestinal bleeding.

**Patient concerns:**

An 8-year-old Chinese boy was transferred to the pediatric intensive care unit of our hospital with an emergency occurrence of purpura, severe hematemesis, large bloody stools, and sharp abdominal pain, and complained of abdominal pain and rash 2 weeks prior.

**Diagnosis:**

The patient had purpura with lower limb predominance, abdominal pain, and gastrointestinal bleeding. Immunofluorescence microscopy of histological sections showed granular and lumpy IgA focal deposition in the blood vessel walls. He was diagnosed with HSP.

**Interventions:**

Initially, he was treated with methylprednisolone, posterior pituitary injection, somatostatin, and hemocoagulase, together with the infusion of large blood products. Postoperatively, he was administered nasal continuous positive airway pressure -assisted ventilation, anti-infection treatment, albumin transfusion, platelet transfusion, abdominal drainage, methylprednisolone, fluconazole anti-fungal treatment, and wound dressing.

**Outcomes:**

There was no evidence of rebleeding, abdominal pain, or purpura at the 2-month follow-up assessment.

**Lessons:**

Abdominal HSP should be alert to gastrointestinal perforation when using hormone therapy.

## 1 Introduction

Henoch-Schoenlein purpura (HSP) is a systemic small-vessel vasculitis that commonly occurs in children. It is characterized by non-thrombocytopenic palpable purpura located largely in dependent parts such as the lower extremities and buttocks, arthralgia/arthritis, bowel angina, and hematuria/proteinuria.^[1,2]^ Gastrointestinal involvement, including colicky abdominal pain, vomiting, and gastrointestinal bleeding, occurs in approximately 50% to 80% of children with HSP.^[3,4]^ About 18% of children experience gastrointestinal bleeding, with most of them having positive fecal occult blood associated with melena and, more rarely, hematemesis.^[3]^ HSP with severe alimentary tract hemorrhage is rare, while intestinal perforation in HSP, although rare, is life-threatening.

## 2 Case presentation

An 8-year-old Chinese boy with no specific family or psychosocial history was admitted to our hospital with an emergency occurrence of purpura, severe hematemesis, large bloody stools, and sharp abdominal pain. His symptoms had begun 2 weeks prior to admission, with abdominal pain and an erythematous pinpoint rash on his arms. The rash developed from his upper limbs (opisthenar and arms) to his lower limbs. Laboratory examination in the outpatient clinic revealed no abnormalities in indicators such as white blood cell count, platelet count, and hemoglobin. After oral medication (specific unknown), his abdominal symptoms began to reduce, as did the purpura. Ten days prior, the rash progressed mostly on the legs, and he experienced deeper abdominal pain in the form of persistent pain with abdominal gassiness and emesis. He was admitted to another hospital with a diagnosis of HSP. Seven days prior, he had melena. After fasting and fluid replacement, there was no obvious improvement in the symptoms. Half a day prior, his face and lips appeared pale, and he had increased abdominal pain, accompanied by numerous bloody stools and hematemesis. He was immediately treated with methylprednisolone, posterior pituitary injection, somatostatin, and hemocoagulase, together with the infusion of large blood products (Table 1). At this stage, the patient still produced large amounts of blood in the vomit and stool. His vital signs were extremely unstable, and he was transferred to the pediatric intensive care unit of our hospital. The patient had purpura with lower limb predominance and abdominal pain (consistent with one of the following 4 criteria: abdominal pain, histopathology, arthritis or arthralgia, and renal involvement), and was diagnosed with HSP.^[1,4]^

**Table 1:** Summaries of clinical data, treatment, and investigations in this case report.

Admission time	10 d before admission	7 d before admission	0.5 d Before admission	1 d	2 d	3 d	4 d surgery	5 d	6 d	7 d	8 d
Abdomen pain	Yes	More	Most	Yes	Yes	Unconsciousness	Sedation	Sedation	Yes	Yes	
Rash	Yes	Yes	Yes	Yes	Yes						
Abdomen Gassiness	Yes	Yes	More	Yes	Yes	Unconsciousness	Sedation	Sedation			
Black stools		Yes									
Blood stools			Yes	Yes	Yes	Yes	Yes	Yes	Yes		
Hematemesis			Yes	Yes	Yes	Yes					
Methylprednisolone (mg)			Yes	200	700	700		40	40	40	40
Cryoprecipitation transfusion (U)			10.0	6.0	10.5	19.5	7.5				
Concentrated RBCs transfusion (U)			14.0	14.0	45.0	53.5	11				
Plasma transfusion (mL)			1600	950	3400	5000	1000	200			
Platelet transfusion (U)				1.0	8.0	2.0			1.0		
Albumin transfusion (g)				40	100	110	20		10	20	30
Immunoglobulin transfusion (g)					10		5	5	5		
Hemoglobin minimum (g/L)				66	39	41	99	103	105	115	124
Platelet minimum (×10^9^/L)	Normal	Normal	—	13	11	11	38	29	99	127	115
Albumin minimum (g/dL)				1.0	1.6	2.3	3.6		3.1	3.2	
APTT (s)				119.2	113.5	133.8	46.8	39.6			
Plasma prothrombin time (s)				19.7	21.3	22.5	12.5	12.1			
Plasma fibrinogen assay (g/L)				0.56	0.46	0.5	1.62	1.25			

APTT = activated partial thromboplastin time, RBC = red blood cell.

**Figure 1:**
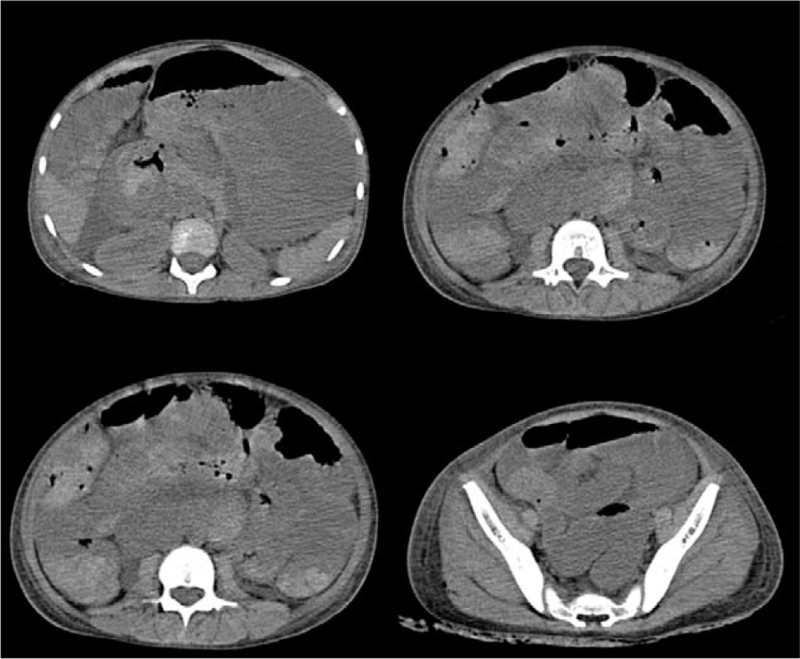
Abdominal CT scanning revealed changes in the stomach, arc-shaped fluid and gas shadows, and peritoneal effusion. CT = computed tomography.

**Figure 2:**
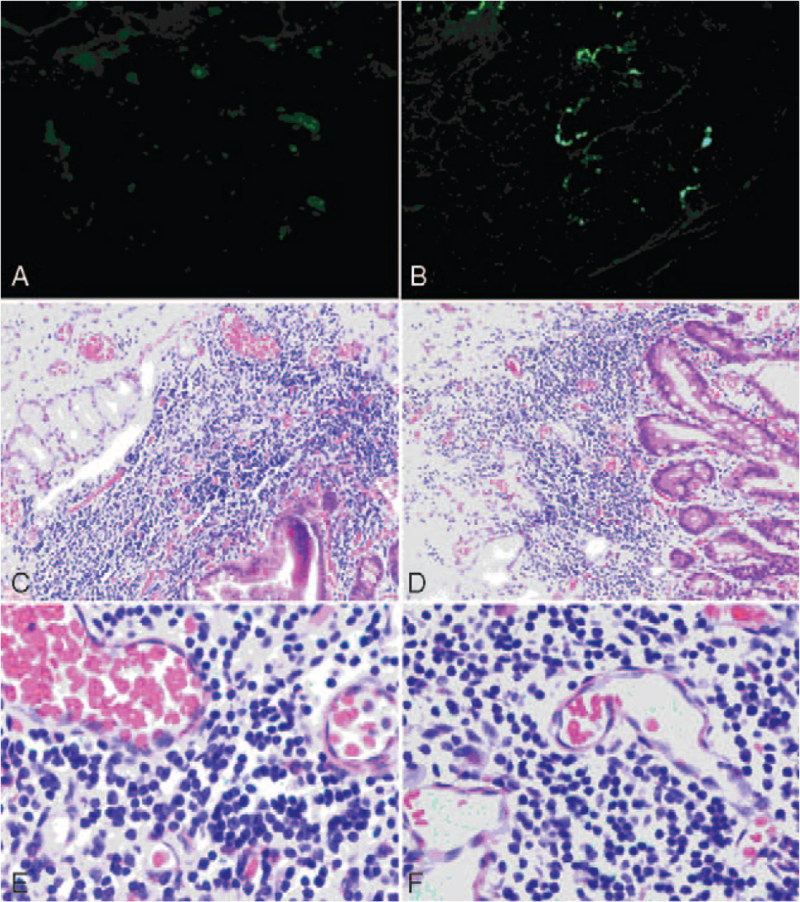
Immunofluorescence of paraffin sections: IgA ++ (A), C3 +++ (B), showing granular and lumpy focal deposition in the blood vessel walls. Histopathological findings (hematoxylin-eosin staining) (C–F): showing mucosal and submucosal interstitial vasodilatation and focal bleeding, perivascular, and interstitial neutrophilic infiltration and leukocytoclasia.

On admission, he had severe anemia, with a blood pressure of 76/44 mm Hg, pulse of 135 beats/min, and respiratory rate of 25 breaths/min. There was also a palpable purpura rash on both legs, tenderness in the abdomen, and absence of bowel sounds. Routine blood examination revealed a hemoglobin level of 70 g/L, white blood cell count of 11.48 × 10^9^/L, and platelet count of 13 × 10^9^/L. The liver function test showed albumin 10 g/L. The serum electrolytes showed sodium 140 mmol/L, potassium 2.92 mmol/L, chloride 112.1 mmol/L, and bicarbonate 15.9 mmol/L (Table 1) with coagulopathy. Therefore, HSP complicated with an alimentary tract hemorrhage was diagnosed. He was immediately treated with intravenous methylprednisolone (2 mg/kg per day), infusion of blood products, and somatostatin but after 2 days after treatment (day 3), he still had massive gastrointestinal hemorrhage, poor circulation, a reduced level of consciousness, and unstable vital signs. Abdominal ultrasonography and plain abdominal radiography revealed no enterobrosis. The patient was also evaluated by pediatric surgeons, and the continuation of medical treatment was suggested. However, his condition deteriorated, and he needed to rely on a life-supporting blood transfusion. He developed signs of peritonitis with abdominal distension. Gastrointestinal tract perforation was suspected. A contrast-enhanced computed tomography (CT) scan of the whole abdomen revealed curved liquid samples and gas shadows on the left and front of the stomach, supporting the possibility of gastrointestinal tract perforation (Fig. 1). On day 4, an emergency laparotomy was performed. The surgery revealed that the anterior wall of the descending duodenum was thinner with a 1 cm × 1 cm diameter perforation, and the posterior wall had a 3 cm × 1 cm ulcer, which had penetrated into the peritoneum of the pancreas and experienced active bleeding from the broken end of the vessel. The intraoperative hemorrhage volume was approximately 3000 mL, including intragastric bleeding and intestinal blood clots. Pathological examination indicated chronic inflammation of the intestinal wall mucosa in the descending part of the stomach and duodenum, with hemorrhage, ulceration, interstitial congestion, and edema. Immunofluorescence microscopy of paraffin sections showed granular and lumpy IgA focal deposition in the blood vessel walls (Fig. 2). Postoperatively, he was administered nasal continuous positive airway pressure-assisted ventilation, anti-infection treatment, albumin transfusion, platelet transfusion, abdominal drainage, methylprednisolone, fluconazole antifungal treatment, and wound dressing. The general symptoms gradually improved, vital signs were stable, and abdominal pain was relieved. The drainage tube was assessed after discharge, the abdominal CT was repeated after 1 month and the duodenostomy tube was removed 50 days after surgery. There was no evidence of rebleeding during the 2-month follow-up assessment.

## 3 Discussion

The patient was diagnosed with HSP, mainly involving the gastrointestinal tract. He developed a large amount of melena and hematemesis on day 7 after the onset of disease despite receiving corticosteroid and blood infusion therapy. The condition did not improve and even developed hemorrhagic shock. Duodenal bulb ulcers and intestinal perforation lesions were eventually discovered during laparotomy. HSP-associated intestinal perforation is a rare complication, with an estimated prevalence of 0.38%.^[5]^ HSP is the most common vasculitis disorder, especially involving small blood vessels. The gastrointestinal tract is characterized by hemorrhage, hydrops, anabrosis, and ulcers.^[6,7]^ The pathogenesis of bowel perforation may result from vasculitis-induced thrombosis, leading to ischemia and consequent necrosis of the bowel wall.^[8]^

The use of corticosteroids to treat children with gastrointestinal bleeding due to HSP remains controversial. Early corticosteroid therapy in HSP patients relieves abdominal pain within 24 hours and reduces the risk of persistent renal disease.^[9]^ In contrast, some studies have demonstrated^[10]^ that corticosteroids may increase the risk of bowel perforation by increasing gastric acid and pepsin secretion and reducing gastrointestinal mucosal resistance, which may induce or exacerbate gastric and duodenal ulcers, and may further cause gastrointestinal bleeding or perforation. In our case, we postulated that vasculitis-induced mucosal ischemia was the cause of the ulcer, which subsequently perforated and hemorrhaged, leading to diffuse peritonitis, and hemorrhagic shock. Histopathological sections revealed classic leukocyte fragmentation vasculitis and immunoglobulin A deposition in the capillaries, supporting the diagnosis of allergic purpura. Surgery revealed a bleeding ulcer and intestinal perforation. We inferred that corticosteroid use may induce or exacerbate duodenal ulcers, inhibit epithelial cell regeneration and granulation tissue formation, inhibit mucosal renewal, and increase the risk of intestinal bleeding and perforation.^[11,12]^ in addition, the anti-inflammatory and analgesic properties of corticosteroids may mask the symptoms of gastroduodenal ulcers and intestinal perforation, thereby delaying the diagnosis and leading to serious adverse consequences. When the condition changed on the third day after admission, abdominal CT scanning was performed. The diagnostic findings of this case on the CT scan included a stomach gas-liquid shadow and peritoneal effusion. However, frequent findings of intestinal ischemia include thickening of the intestinal wall, dilatation of the lumen, and intra-abdominal fluid, which cannot be differentiated from other diseases, and it is difficult to locate the lesion.^[7,13]^ Therefore, when HSP with gastrointestinal bleeding does not improve but worsens after corticosteroid treatment, attention should be paid to the potential complications of gastrointestinal ulcers and intestinal perforation. Gastrointestinal endoscopy should be completed as soon as possible, and laparotomy should be performed to detect the lesions. Early diagnosis and clear etiology, together with timely and appropriate treatment, are required to reduce morbidity and mortality in these patients.

## Author contributions

**Formal analysis:** Hongyan Tang.

**Investigation:** Wei Du.

**Project administration:** Yiyi Ding.

**Visualization:** Wei Du.

**Writing – original draft:** Shuo Wang.

**Writing – review & editing:** Hongyan Tang, Yiyi Ding.
